# A randomized phase-I pharmacokinetic trial comparing the potential biosimilar tocilizumab (QX003S) with the reference product (Actemra^®^) in Chinese healthy subjects

**DOI:** 10.1080/07853890.2021.1887925

**Published:** 2021-02-25

**Authors:** Hong Zhang, Xiaojiao Li, Jingrui Liu, Cuiyun Li, Min Wu, Xiaoxue Zhu, Jixuan Sun, Min Fang, Yanhua Ding

**Affiliations:** a Phase I Clinical Research Center, The First Hospital of Jilin University, Jilin, China; b Qyuns Therapeutic Co. Ltd., Taizhou City, Jiangsu Province, China

**Keywords:** Tocilizumab, biosimilar, immunogenicity, pharmacokinetics, inter-subject variability

## Abstract

**Purpose:**

QX003S is a biosimilar candidate for the reference tocilizumab, Actemra®. We investigated the tolerance, variability, and pharmacokinetics (PK) of QX003S biosimilar in healthy Chinese male subjects.

**Design:**

A randomised, double-blind, two-arm, parallel study was performed to examine the bioequivalence of QX003S (8 mg/kg) with that of Actemra® as a reference drug.

**Results:**

QX003S (*N* = 40) and Actemra® (*N* = 40) groups exhibited similar PK properties. The inter-subject variability ranged from 14.95% to 18.78%. The 90% confidence intervals of the ratios for C_max_, AUC_0–t_ andAUC_0–∞_ in both groups were within the range of 80–125%. After administration, the number of subjects who tested positive for anti-drug antibodies (ADA) in the QX003S group and Actemra® groups was 6 (14.3%) and 14 (34.1%), respectively. Adverse reactions occurred in 100% and 97.6% subjects in the QX003S and Actemra® groups, respectively. The most common adverse reactions were decrease in fibrinogen level and neutrophil and leukocyte counts.

**Conclusion:**

The PK characteristics and immunogenicity exhibited by QX003S were similar to that of the reference product, Actemra®. The safety profile was similar in the two treatment groups with mild-moderate adverse effects.

**Trial Registration**
The trial is registered at Chinese Clinical Trial website (http://www.chinadrugtrials.org.cn/index.html#CTR20190002)

**Key points**
- This was the first clinical report of a new proposed tocilizumab biosimilar, QX003S.
- This phase-I randomized, controlled study compared pharmacokinetics, variability,immunogenicity, and safety of QX003S vs. the approved tocilizumab product (Actemra@).
- The results demonstrate bioequivalence between BAT1806 and the reference products (Actemra@), as well as comparable immunogenicity, safety and tolerability profiles.

## 1. Introduction

Biological products are large and complex molecules, usually derived from living cells. Due to the molecular complexity and multifaceted production process, the characteristics of biosimilars differ from those of the traditional small-molecule drugs [1–3]. Despite significant therapeutic advances, biologic therapies, such as monoclonal antibodies, are expensive with limited global access [4].

The US Food and Drug Administration (FDA), the European Medicines Agency (EMA), and the National Medical Products Administration (NMPA) have emphasized a step-by-step approach for the development of biosimilars [1]. Biological functional similarity is assessed in the first step, followed by the assessment of pharmacokinetic (PK) and pharmacodynamic (PD) characteristics; finally, the clinical similarity (efficacy, safety, and immunogenicity) is assessed using the same approved dose and pathway as the reference product [1–3].

Tocilizumab binds to soluble and membrane-bound interleukin (IL)-6 receptors and through these receptors inhibits IL-6-mediated signal transduction. IL-6 is a multipotent pro-inflammatory cytokine produced by a variety of cell types, including T and B cells, lymphocytes, monocytes, and fibroblasts. Synovial cells and endothelial cells also produce IL-6, which induces the inflammatory process in the joints (e.g. rheumatoid arthritis) [5–6]. Tocilizumab is effective against rheumatoid arthritis, giant cell arteritis, and multi-joint juvenile idiopathic arthritis. In a previous study, tocilizumab reduced the likelihood of progression to the composite outcome of mechanical ventilation or deathin hospitalised patients with Covid-19 pneumonia; however, it did not improve survival of these patients. Tocilizumab is currently under investigation as a potential treatment for COVID-19, with initial contradictory evidence [7].

Consequently, tocilizumab biosimilars have been actively developed around the world, including in China. Tocilizumab biosimilars (QX003S) have the same primary structure, post-translational modification, biochemical characteristics, and biological functions as the reference product, and in addition, these similarities have been tested in mice and monkeys (data not published). All *in vivo* studies justify the clinical development of QX003S.

PK studies in humans are essential to demonstrate the bioequivalence of biological analogues and reference products [8]. Herein, we conducted a single-dose PK study in healthy Chinese male subjects to evaluate the bioequivalence between QX003S and Actemra@ as the reference product. Use of healthy subjects helps avoid the potential confounding influence of factors such as comorbid diseases and concomitant therapies. The therapeutic dose of the reference drug used in previous studies is 4–8 mg/kg [9–10]. In this study, a dose of 8 mg/kg was used, based on earlier clinical trial plans of the sponsor.

In this study, the PK profiles of the QX003S with Actemra@ were analysed and compared. In addition, the tolerability, safety, and immunogenicity of QX003S were assessed.

## 2. Methods

### 2.1. Study design and subjects

This phase-I study was conducted at the Clinical Research Centre of the First Hospital of Jilin University between 14 March 2019 and 18 September 2019 (Chinese Clinical Trial Registry, Registration No. CTR20190002). The study protocol was approved by the ethics committee of the hospital. The study complied with the guidelines of the Declaration of Helsinki and the International Conference on Harmonisation (ICH) Good Clinical Practice (GCP). Written Informed consent was obtained from all subjects prior to their enrolment.

**Figure 1.**
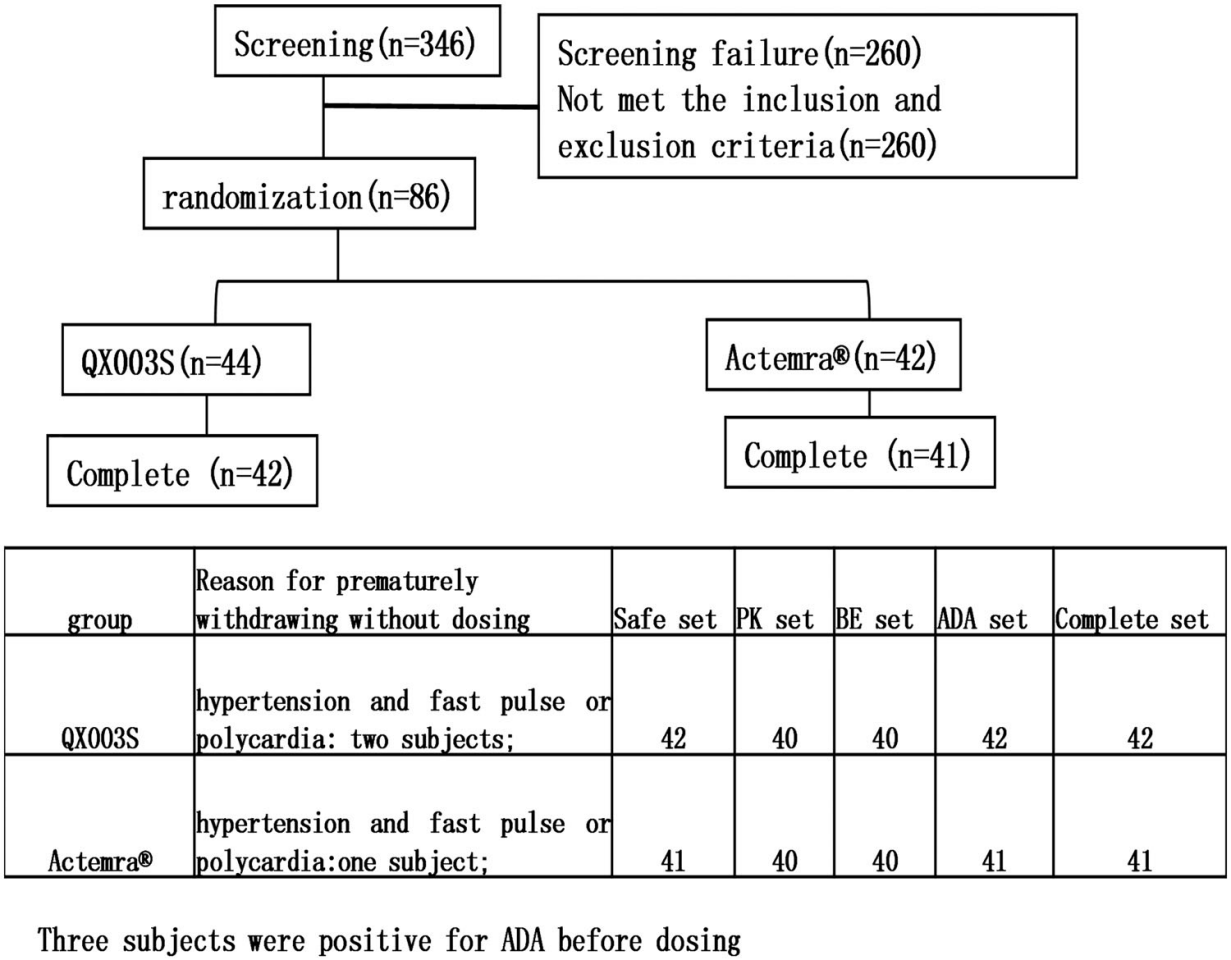
Flow chart of the study.

This was a randomized, double-blind, single-dose, two-arm, parallel comparison study to evaluate the PK, safety, and immunogenicity of QX003S and Actemra@ in healthy Chinese male subjects. Overall, 86 eligible subjects were randomly allocated in a 1:1 ratio to receive a single intravenous drip of 8 mg/kg QX003S or Actemra@. Subjects were stratified into two groups based on body weight (50 to < 67.5 kg and ≥ 67.5 to ≤ 85 kg). Individuals in each of the pre-specified groups were equally assigned to the two treatment groups through randomization (Figure 1).

Sentinel staggered administration was used in this study. Subjects were administered the investigational product (IP) in a staggered cohort: the first and second cohort consisted of two subjects and four subjects, respectively. For safety evaluation, each subject was required to stay at the study centre for at least 96 h after the administration. Based on sentinel safety results, the principal investigator determined whether the subsequent subjects would be monitored in sentinel mode or in routine follow-up mode. All subjects were followed up for 57 days.

The main inclusion criteria were as follows: (1) healthy men in the age group of 18–50 years; (2) body mass index: 18.0–28.0 kg/m^2^; (3) body weight: 55–85 kg; and (4) normal test outcomes or clinically unremarkable results of routine blood and urine routine investigations including hepatic and renal function tests during enrolment.

The exclusion criteria were as follows: (1) history of clinically significant diseases; (2) C-reactive protein (CRP) levels 1.5 times higher than the upper limit of the normal range; and (3) positive results of T-SPOT® assay or TB interferon-γ-release assay.

All subjects received a single intravenous infusion of the IP (8 mg/kg) administered over a period of 60 min (±6 min). All subjects were randomly allocated to one of the following two groups in a 1:1 ratio in each of the pre-specified weight intervals: QX003S (Jiangsu Quanxin Biomedicine Co. Ltd; Batch number: F20180801); Actemra@ (Chugai Pharmaceutical Company [Japan]; Batch number: B2063B15).

Screening was performed 14 to 2 days prior to the date of administration. All qualified subjects entered the clinical research unit a day prior to the administration of biosimilars. Subjects were required to fast for at least 8 h before administration and were randomly assigned to either the test drug (QX003S) or reference drug group.

### 2.2. PK evaluations

Blood samples were collected for PK analysis at different time-points: 1 h before administration (before administration) to 1344 h after the initial infusion (day 57). Serum tocilizumab levels were determined by enzyme-linked immunosorbent assay (ELISA) at the Junke Zhengyuan (Beijing) Pharmaceutical Research Co. Ltd. (Supplement material). PK parameters were determined by non-compartmental analysis model. The concentration–time data included the maximum observable serum concentration (C_max_), clearance (CL), half-life (t_1/2_), the volume of distribution (Vz), and area under the curve (AUC) from zero to the final quantifiable concentration (AUC_0–t_) and to infinity (AUC_0–∞_). The actual sampling times were used for PK analyses. An internally validated software system, Phoenix WinNonLin® v8.0 (Pharsight Corporation, Certara, L.P., Princeton, New Jersey, USA), was used to determine PK parameters.

### 2.3. Immunogenicity evaluations

Blood samples collected at 1 h before and on 15, 29, 43, and 57 days after drug administration were analysed for the presence of anti-drug antibodies (ADAs) using electrochemiluminescence immunoassay (ECLIA). Subjects who test ADA-positive, those who develop antibody-related adverse reactions, or those with significantly abnormal PK value are required to be further examined for the presence of neutralising antibodies (NAbs). NAb test was not performed in this study because the above conditions were not met.

### 2.4. Safety evaluation

Physical examination, assessment of vital signs, electrocardiogram, and routine laboratory investigations were performed to monitor adverse events (AEs) according to the National Cancer Institute Common Terminology for Adverse Events (CTCAE;V.4.03). Subjects who showed AEs were monitored until they reached normal or acceptable stability (as assessed by the principal investigator and sponsor) or were lost to follow up.

### 2.5. Estimation of the sample size

According to the recent FDA guidelines, the geometric mean ratio (GMR) was set at 95% to achieve 90% power (1 − β) at a significant level (two-sided α = 5%). Inter-subject variability (inter-CV) is expressed by the coefficient of variation (CV). NQuery 8.3.0.0 (Boston, USA) software was used to determine the sample size (initial: 68; inter-CV for tocilizumab: 24%) [9]. The final sample size was 86, allowing for a 20% drop-out rate.

### 2.6. Statistical analysis

After logarithmic transformation of PK parameters C_max_, AUC_0–t_, and AUC_0–∞_, the least square method was used for analysis of variance. Bioequivalence inferences were drawn if the 90% confidence intervals (CIs) were found to be within the range of 80–125%. PK analysis was performed using the PK analysis set. The safety analysis set included subjects who were administered the study drug. Descriptive statistical estimates of PK parameters and demographic data were calculated. Between-group differences were assessed using the Chi-squared test for categorical variables, *t*-test for normally distributed continuous variables, and Wilcoxon rank test for non-normally distributed variables. All statistical analyses were performed using SAS 9.4 (SAS Institute Inc., Cary, NC, USA).

## 3. Results

### 3.1. Subjects

The assigned drugs were administered to 83 of the 86 enrolled subjects and included in the safety analysis (Figure 1). One additional subject was included in the QX003S group, whereas one subject was removed from the Actemra® group due to weight stratification. Therefore, the QX003S group comprised of 44 subjects.

Before dosing, two and one subjects in the QX003S and Actemra® groups, respectively, had hypertension and fast pulse rate or polycardia; these subjects were excluded from the study. Before dosing, two and one subjects in the QX003S and Actemra® groups, respectively, were ADA positive;these were excluded from the study. The final per-protocol analysis population included in the safety, PK, BE, and immunogenicity (ADA) analysis set comprised of 83, 80, 80, and 83 subjects, respectively (Figure 1). The demographic and baseline characteristics of the per-protocol population and the two treatment groups were comparable (*p* > .05, Table 1).

**Table 1.** Demographic and baseline characteristics.

	QX003S group	Actemra® group	Total	
	(*n* = 44)	(*n* = 42)	(*n* = 86)	*p* Values
Age (year), mean (SD)	35.5 (8.88)	36.1 (8.51)	35.8 (8.66)	.75
Ethnicity (Han, n [%])	41 (93.2)	40 (95.2)	81 (94.2)	.68
Weight (kg), mean (SD)	67.45 (9.423)	66.21 (7.621)	66.84 (8.563)	.5
BMI (kg/m^2^), mean (SD)	23.306 (2.5834)	23.234 (2.5210)	23.271 (2.5383)	.89

BMI; body mass index; SD: standard deviation.

### 3.2. PK evaluation

**Figure 2.**
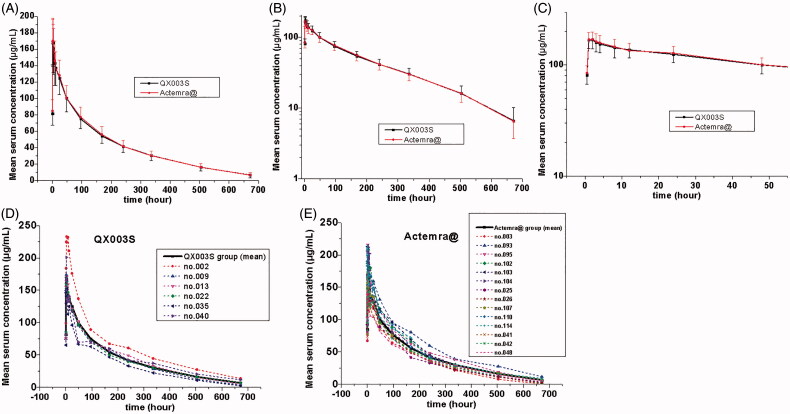
Serum drug concentration–time profile of tocilizumab. Mean values (A); log10 mean values (B); log10 mean values within 0–48 h (C); ADA-positive individuals in the QX003S (D) and Actemra@ (E) groups.

The mean serum concentration–time curve of tocilizumab and its biosimilar decreased with multiphase mode. A rapid decline immediately after the infusion was followed by a slow elimination phase, and, subsequently, by a slightly faster elimination phase at low concentrations (Figure 2). The non-compartmental analysis model showed slow clearance, longer t_1/2_, and small Vz of tocilizumab and its biosimilar. The median T_max_ values were equivalent between the two groups and these were achieved 1.8 h after the intravenous infusion.

The mean value of t_1/2_ between the test drug and the reference drug was between 160.8155 and 159.9160 h, indicating comparability. The total clearance rate (CL) and Vz values were also similar in the two groups. The differences between the mean concentration–time curve, mean C_max_, AUC0-t, AUC0-∞ estimation, and inter-CVs were similar (*p* > .05); the coefficient of variation ranged from 14.95% to 18.78% (Table 2, Figure 2).

**Table 2.** Pharmacokinetic parameters of tocilizumab in each group (Mean ± SD [CV%] or median [min, max]).

	QX003S group (*n* = 40)	Actemra® group (*n* = 40)	*p* Values	GMR (90% CI)	GMR (90% CI)^a^	Re-estimated size
T_max_*(h)	1.8 (1-4)	1.8 (1-4)	>.05			
C_max_ (μg/mL)	178.8 ± 28.90 (16.16)	178.2 ± 27.43 (15.39)	.92	1 (0.95, 1.06)	1.02 (0.95-1.09)	40
AUC_0-t_ (h•μg/mL)	27116.0941 ± 4466.9216 (16.47)	27446.4185 ± 4103.9469 (14.95)	.73	0.9859 (0.9311,1.0439)	0.9827 (0.9197-1.0500)	40
AUC_0-∞_ (h•μg/mL)	28806.7645 ± 5411.8467 (18.78)	29039.7894 ± 4641.0883 (15.98)	.83	0.9878 (0.9272, 1.0524)	0.9787 (0.9098-1.0529)	52
t_1/2_ (h)	160.8155 ± 35.2772 (21.93)	159.9160 ± 29.0054 (18.13)	.90			
CL (L/h)	0.0192 ± 0.0031 (16.06)	0.0185 ± 0.0025 (13.68)	.26			
V_d_ (L)	4.3578 ± 0.7660 (17.57)	4.2322 ± 0.7760 (18.33)	.46			

*Median [min, max]; ^a^QX003S/ Actemra® after excluding subject with ADA positive after dosing.

The PK parameters were comparable in the QX003S and Actemra@ groups. The ratio of geometric least-squares means for the QX003S versus Actemra@ were 1, 0.9859, and 0.9878 for C_max_, AUC_0-t_, and AUC_0-∞_;the 90% CI was 0.9272–1.06. The 90% CIs of the C_max_, AUC_0–t_, and AUC_0–∞_ were within the predefined bioequivalence limit, ranging from 80.00% to 125.00%. A larger inter-CV indicated a broader 90% CI. The sample size was re-estimated on the basis of the results of bioequivalence analysis (GMR and inter-CV), which decreased to a number less than the enrolment size (Table 2).

### 3.3. Immunogenicity evaluation

Before dosing, two and one subjects in the QX003S and Actemra® group respectively, were ADA positive. After dosing, 6 (14.3%) subjects in the QX003S group and 14 (34.1%) subjects in the Actemra® group tested positive for ADA. The ADA-positive rates were found to increase over a period of time, especially by days 43 (1008 h) and 57 (1344 h). Nevertheless, the drug concentration was less than the lower limit of quantitation (LLOQ) during that period. ADA-positivity rates were similar in the two groups at 15 to 29 days after drug administration (6.7–7.1%). However, at 43 and 57 days after drug administration, the positivity rates in the QX003S group were relatively lower than those in the Actemra@ group; however, the between-group differences in this respect were not statistically significant at any of the time-points (*p* > .05, Table 3).

**Table 3.** Summary of immunogenicity (anti-drug antibody) assessment (number [%] of subjects with positive antibodies).

Time (day)	QX003S group (*n* = 42)	Actemra® group (*n* = 41)	*p* Values
Pre-dose	2 (4.88)	1 (2.44)	.57
15	0 (0)	0 (0)	NA
29	0 (0)	1 (2.44)	.30
43	3 (7.32)	5 (12.2)	.43
57	8 (19.51)	13 (31.71)	.18

NA: Not applicable.

**Table 4.** Adverse reactions (number of reactions, the number [%] of subjects, more than 4%).

	QX003S group (*n* = 42)		Actemra® group (*n* = 41)		
	n (%)	[number of reactions]	n (%)	[number of reaction]	*p* Values
Total	42 (100)	134	40 (97.6)	148	0.30
Fibrinogen decreased	38 (90.5)	40	34 (82.9)	34	0.31
Reduced neutrophil counts	30 (71.4)	39	24 (58.5)	32	0.21
Reduced leukocyte count	24 (57.1)	28	19 (46.3)	26	0.32
Elevated serum bilirubin	6 (14.3)	6	3 (7.3)	5	0.30
Elevated alanine aminotransferase	0 (0)	0	5 (12.2)	6	0.01
Elevated aspartate aminotransferase	0 (0)	0	4 (9.8)	6	0.03
Reduced lymphocyte count	1 (2.4)	2	2 (4.9)	2	0.54
Urine leucocyte positive	0 (0)	0	2 (4.9)	2	0.14
Oropharyngeal pain	2 (4.8)	2	3 (7.3)	3	0.62
Cough	2 (4.8)	2	2 (4.9)	2	0.98
Cough with expectoration	1 (2.4)	1	2 (4.9)	2	0.54
Runny nose	0 (0)	0	3 (7.3)	3	0.07
Stuffy nose	0 (0)	0	2 (4.9)	2	0.14
Hypertriglyceridaemia	5 (11.9)	5	7 (17.1)	7	0.50
Hyperuricemia	1 (2.4)	1	2 (4.9)	3	0.54
Diarrhea	2 (4.8)	2	1 (2.4)	1	0.57
Oral mucositis	0 (0)	0	2 (4.9)	2	0.14

The serum concentration-time curves of QX003S and Actemra@ for ADA-positive and ADA-negative subjects were found to be similar (Figure 2). Sensitivity analysis of bioequivalence was performed after exclusion of 20 subjects who tested positive for ADA. The 90% CIs for the comparisons of C_max_ and AUC were within the predefined range of bioequivalence limits of 80.00%–125.00% (Table 2). Therefore, overall, ADA-positivity rates were similar in the two groups.

### 3.4. Safety evaluation

No serious AEs (SAEs), deaths, or discontinuations due to AEs were observed. In this study, 282 adverse reactions occurred in 82 (98.8%) subjects. A total of 134 adverse reactions in 42 (100%) subjects were recorded in the QX003S group, while148 adverse reactions in 40 (97.6%) subjects were recorded in the Actemra® group. The incidence of adverse reactions was comparable in the two groups (Table 4). The adverse reactions with an incidence greater than 5% in the QX003S and Actemra® groups, respectively, were as follows: decreased fibrinogen level (90.5% vs 82.9%), decreased neutrophil count (71.4% vs 58.5%), decreased white blood cell (WBC) count (57.1% vs 46.3%), increased bilirubin (14.3% vs 7.3%), and hypertriglyceridaemia (11.9% vs 17.1%). The severity of most adverse reactions was between grade I and II. The incidence rates of elevated alanine aminotransferase and elevated aspartate aminotransferase level in the QX003S group were lower than those in the Actemra® group (0% vs. 12.2%, *p* = 0.01; 0% vs. 9.8%, *p* = 0.03); this indicated a lesser effect of QX003S on liver enzyme levels than the reference product. The incidence of other adverse reactions was comparable in the two groups (*p* > 0.05).

A total of 20 (24.1%) subjects in the two groups experienced 26 grade III–IV adverse reactions. Thirteen (31.0%) subjects in the QX003S group developed 16 adverse reactions, and seven (17.1%) subjects in the Actemra® group developed 10 adverse reactions. The incidence in the QX003S vs Actemra® groups was comparable: decreased neutrophil count (28.6% vs 12.2%), decreased WBC count (7.1% vs 7.3%), decreased fibrinogen (2.4% vs 2.4%), and increased alanine aminotransferase (0 vs 2.4%). All grade III–IV adverse reactions recovered spontaneously without treatment. Very few Grade I-II adverse reactions required drug therapy, such as cefuroxime axetil, levofloxacin, and glycyrrhizin.

There was no association between ADA development and adverse reactions in this study. None of the subjects developed clinically significant or serious hypersensitivity, anaphylaxis, or injection-site reaction after IP administration, except Subject no.105 of the QX003S group who developed ecchymia and mild tenderness at the injection site 48 h after administration; this subject showed spontaneous recovery on day 8 without any treatment. Subject no.001 of the Actemra® group showed bruising at the injection site at 12 h after administration without any tenderness; this subject also showed spontaneous recovery on day 22 without any treatment. All adverse reactions were reported to the Institutional Review Board of The First Hospital of Jilin University.

## 4. Discussion

This single-dose, phase-I study demonstrated the bioequivalence of QX003S and Actemra@ when administered as intravenous infusion at a dose of 8 mg/kg. The results of ANOVA showed that the 90% CIs of the geometric mean ratios of C_max_ and AUC in the two treatment groups ranged from 92.72%–106%, which was within the predefined bioequivalence intervals of 80% to 125%. Other PK parameters of T_max_ and t_½_ were also similar between the two treatment groups. QX003S and Actemra@ showed a similar safety and immunogenicity profile. No serious AEs were reported; all adverse reactions were mild or moderate in severity, and no local reactions were reported except in two subjects. This indicated that the two products were well tolerated in this population of healthy subjects. The above results justify the use of the biosimilars in the next phase clinical studies [1–3].

The pharmacokinetic behaviour of tocilizumab is different from the small-molecule pharmacokinetic behaviour in that it has limited vascular permeability, neonatal Fc receptor circulation, and more frequent receptor-mediated nonlinearity. Its distribution and clearance (CL) are consistent with target-mediated drug disposition (TMDD) [11]. On average, C_max_ of tocilizumab decreased approximately 55% in the first 96 h. Subsequently, a slow elimination phase was observed between 96 and 336 h, followed by a relatively fast elimination between 336 and 672 h (Figure 2). In this study, QX003S at a dose of 8 mg/kg [mean weight of the subjects: 67.45 kg, dosage: 539.6 mg (4 × 67.45)] showed a lower clearance and displayed a longer t_1/2_ (160.8155 vs 39.9 h) than tocilizumab 162 mg (Roche Products Limited, Welwyn Garden City, UK), more exposure (AUC ratio of QX003S vs tocilizumab 162 mg equal to 6.39) than dose ratio (539.6:162 = 3.33), similar T_max_ and dose ratio of C_max_ with tocilizumab 162 mg, which have been evaluated in other phase-I studies in healthy subjects (Supplement Table 1) [12].

Population pharmacokinetic analyses in any patient population tested so far indicate no relationship between apparent clearance and the presence of anti-drug antibodies [9,13]. Similarly, ADA had no effect on drug concentration or bioequivalence results in this study (Figure 2, Table 2). In population PK analysis, body weight was identified as a significant covariate impacting the pharmacokinetics of tocilizumab. When administered intravenously on mg/kg basis, individuals with body weight ≥100 kg are predicted to have higher exposures than individuals with body weight <100 kg. Therefore, weight stratification was adopted in this study to reduce variation of parameters, although the weight of subjects in this study was <100 kg. The inter-CV of tocilizumab was small (less than 18.7867%); therefore, the sample size in future studies can be reduced to 52 subjects (26 subjects per arm) [14].

**Figure 3.**
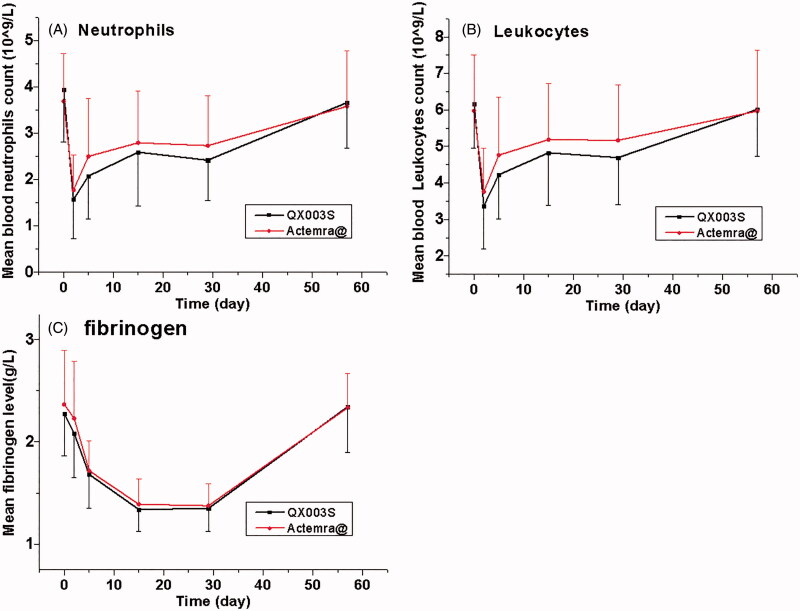
Absolute values of neutrophil, leukocyte counts, and fibrinogen level over time. Data presented as mean ± standard error of the mean.

Notably, the incidence of adverse reactions in the QX003S group was similar to that in the Actemra@ group (100% vs 97.6%); most of these were resolved at the final visit in this study. The most common adverse reactions (incidence of at least 5%) are reported in the label, including upper respiratory tract infections, nasopharyngitis, headache, hypertension, increased ALT level, and injection site reactions [9]. In healthy subjects who were administered ACTEMRA in doses of 2–28 mg/kg intravenously and 81–162 mg subcutaneously, the absolute neutrophil counts decreased to the nadir 3 to 5 days following administration. Thereafter, the neutrophil counts recovered towards baseline in a dose-dependent manner over a period of 9–17 days [12]. Patients with rheumatoid arthritis and GCA exhibited a similar pattern of absolute neutrophil counts following the administration of ACTEMRA [9,15]. Similar to previous reports, the incidence of decreased neutrophil counts and decreased white blood cell counts was indeed very high. Neutrophil counts decreased on day 2 to 5; the mean neutrophil count reached nadir on day 2 in both groups. The mean values returned to baseline by day 57 without any treatment (Figure 3). As described by Nishimoto *et al.* [16], when tocilizumab concentration is maintained above 1 µg/mL, SIL-6R is saturated by tocilizumab leading to complete inhibition of the IL-6 signal; this may affect the distribution of blood cells such as neutrophils and leukocytes. However, these cell counts quickly return to baseline with the drop in the drug concentration.

In clinical studies, RA patients were treated with 4–8 mg/kg intravenous doses or the 162 mg weekly and every other weekly subcutaneous doses of ACTEMRA; the levels of CRP decreased to within the normal range along with changes in the pharmacodynamic parameters (i.e. decrease in rheumatoid factor, erythrocyte sedimentation rate (ESR), serum amyloid A, fibrinogen; and increase in haemoglobin) [9]. In the present study, fibrinogen level decreased in 82.9–90.5% subjects and the mean fibrinogen level reached the nadir on day 15 to 29 in the QX003S and Actemra® groups; these changes are similar to the above changes in pharmacodynamic indices.

No acute or delayed anaphylactic reactions developed in subjects who were ADA positive, indicating that there was no product-specific immunogenicity. We observed no impact of the immunogenic responses of tocilizumab to drug safety and PK in this study similar to previous reports; however, it may still be necessary to closely monitor the immunogenicity of QX003S and Actemra and its impact on their efficacy in further related Phase-III studies with larger population, multiple doses, as well as longer time frame [17–19]. Overall, this study demonstrated the safety and tolerability of QX003S and reference Actemra@.

## Conclusions

This study showed similar PK profile of tocilizumab biosimilar (QX003S) and Actemra@. The tocilizumab biosimilar showed a nearly similar ADA profile and a comparable safety profile versus the reference drug. The inter-CV of tocilizumab was low among Chinese subjects. These data support the clinical development of QX003S as a tocilizumab biosimilar.

## Supplementary Material

Supplemental Material

## Data Availability

The data that support the findings of this study are available on request from the corresponding author, YHD. The data are not publicly available due to their containing information that could compromise the privacy of research participants.
