# Supplementary material for: A randomized phase-I pharmacokinetic trial comparing the potential biosimilar tocilizumab (QX003S) with the reference product (Actemra®) in Chinese healthy subjects
Source: Ann Med. 2021 Feb 25;53(1):375–83. doi: 10.1080/07853890.2021.1887925 (PMC7919877; doi:10.1080/07853890.2021.1887925)
Supplement: Supplemental Material [file IANN_A_1887925_SM9934.docx]

**Supplement**

**1. Methods**

**1.1. Supplement Exclusion criteria**

(1) Use of any biological products within the three-month period immediately preceding the screening or those planning to receive monoclonal antibody drugs within 9 months; (2) blood transfusions within the three-month period immediately preceding the dosing, previous tocilizumab treatment, positive for the anti-tocilizumab or its biosimilar antibody, or positive for HBsAg, HCV antibody, HIV antibody, or treponema pallidum antibody; (3) active infection including acute or chronic infection and local infection; (4) active tuberculosis on the basis of chest x-ray, or a history of tuberculosis, or latent tuberculosis infection or clinically suspected tuberculosis (including but not limited to pulmonary tuberculosis); (5) those who received or planned to receive a live virus vaccine or immunosuppressive agent within 12 weeks before IP administration or during the study. (6) Use of drugs that target tumor necrosis factor or interleukin within 1 year before screening.

**1.2. Collection time points of different safety indicators**

Hematology, biochemistry, and urinalysis tests and markers of myocardial damage: at screening and on days 2, 5, 15, 29, and 57; ECG: screening, at 60 min predose, at 4 h and 12 h after infusion, and on days 2, 3, 5, 8, 15, 29, and 57 after infusion.

**1.3. Pharmacokinetic and immunogenicity evaluations**

Enzyme-linked immunosorbent assay (ELISA) and electrochemiluminescence immunoassay analyzer (ECLIA) methods were used to measure the serum concentrations of tocilizumab, anti-drug antibody (ADA), and [neutralizing](javascript:void(0);) [antibody](javascript:void(0);) (NAb) (Junke Zhengyuan [Beijing] Pharmaceutical Research Co. Ltd.]. First, 4 and 4 mL venous blood samples were collected at each time-point for analyzing serum tocilizumab concentration and immunogenicity, respectively.

Blood samples for PK evaluation were collected 1 h before the initiation of dosing (predose), at 30 min after the start of IP infusion, at the end of infusion (immediately after 60-min infusion), at 2, 3, 4, 8, and 12 h after the start of the infusion, and at 24 h (day 2), 48 h (day 3), 96 h (day 5), 168 h (day 8), 240 h (day 11), 336 h (day 15), 504 h (day 22), 672 h (day 29), 1008 h (day 43), and 1344 h (day 57) after the start of infusion. After collection, the blood samples were allowed to clot for 30 min at room temperature and centrifuged at 1800 g for approximately 15 min at 2–8°C. Serum samples were stored at −70°C prior to further processing. The concentration range was 75.0–2400 ng/mL, and the lower limit of quantification (LLOQ) was 75.0 ng/mL. For the PK analysis, concentrations less than the LLOQ were set to zero. The accuracy of the inter-run assay ranged from 9.1 to 13.9% and was expressed as the percentage relative error for the quality control samples.

**2. Results**

Supplementary Table 1. Comparison of key pharmacokinetic parameters between BAT1806 and group (GeoMean or mean or median (min, max))

| Parameter | QX003S group (N = 40) | Tocilizumab 162 mg [1] | Parameters ratio |
| --- | --- | --- | --- |
| AUC_0-t_ (µg × h/mL) | 27116.0941 | 4300 | 6.31 |
| C_max_ (µg/mL) | 178.8 | 47 | 3.80 |
| T_max_ (h)* | 1.8 (1–4) | 1.5 (1.0–4.0) |  |
| t_1/2_ (h) | 160.8155 | 39.9 | 4.03 |
| CL (L/h) | 0.0192 | 0.0402 | 0.48 |
| Vz (L) | 4.3578 | 2.2 | 1.98 |

*Median [min, max]; the Mean for QX003S in this study and the mean for tocilizumab 162 mg, which were infused over a 1-h period.

[1] Xiaoping Zhang, Angela Georgy, Lucy Rowell. Pharmacokinetics and pharmacodynamics of tocilizumab, a humanized anti-interleukin-6 receptor monoclonal antibody, following single-dose administration by subcutaneous and intravenous routes in healthy subjects. Int J Clin Pharmacol Ther. 2013, 51(6):443-55.
